# Intracellular calcium signal at the leading edge regulates mesodermal sheet migration during *Xenopus* gastrulation

**DOI:** 10.1038/s41598-018-20747-w

**Published:** 2018-02-05

**Authors:** Kentaro Hayashi, Takamasa S. Yamamoto, Naoto Ueno

**Affiliations:** 1 Department of Developmental Biology, National Institute for Basic Biology, 38 Nishigonaka, Myodaiji, Okazaki, Aichi 444-8585 Japan; 2 Department of Basic Biology, School of Life Science, The Graduate University of Advanced Studies (SOKENDAI), 38 Nishigonaka, Myodaiji, Okazaki, Aichi 444-8585 Japan

## Abstract

During the gastrulation stage in animal embryogenesis, the cells leading the axial mesoderm migrate toward the anterior side of the embryo, vigorously extending cell protrusions such as lamellipodia. It is thought that the leading cells sense gradients of chemoattractants emanating from the ectodermal cells and translate them to initiate and maintain the cell movements necessary for gastrulation. However, it is unclear how the extracellular information is converted to the intracellular chemical reactions that lead to motion. Here we demonstrated that intracellular Ca^2+^ levels in the protrusion-forming leading cells are markedly higher than those of the following cells and the axial mesoderm cells. We also showed that inhibiting the intracellular Ca^2+^ significantly retarded the gastrulation cell movements, while increasing the intracellular Ca^2+^ with an ionophore enhanced the migration. We further found that the ionophore treatment increased the active form of the small GTPase Rac1 in these cells. Our results suggest that transient intracellular Ca^2+^ signals play an essential role in the active cell migration during gastrulation.

## Introduction

Gastrulation is one of the most important processes in the early development of a variety of animals. In vertebrates, this dynamic remodelling process is achieved by the coordinated movements of three germ layers, which contribute to the development of various organs in their proper positions in the body. In the experimental vertebrate model *Xenopus laevis*, the gastrulation movements begin with vegetal rotation of the mesodermal sheet^1^. Subsequently, the leading edge mesoderm (LEM), the most vegetal mesoderm region, touches the inner side of the blastocoel roof (BCR). The LEM then shows directional migration toward the anterior end on the fibronectin-rich BCR^2–5^. During this process, axial mesoderm following the LEM undergoes convergent extension, in which cell movements elongate the embryo proper along the anterior-posterior axis and narrow the tissue mediolaterally, eventually forming the rod-shaped notochord^6^. On the other hand, the prechordal mesoderm that follows the LEM cells plays essential roles in head formation^7,8^.

The LEM has an indispensable role in the directional migration of the mesodermal sheet, and disrupting this migration causes severe morphological defects in the embryo, such as abnormal notochord formation and *spina bifida*^9,10^. This anterior tissue migration is thought to be regulated by chemoattractants and/or by cell responses to mechanical signals. As chemoattractants for the directed tissue migration, PDGF^7^ and SDF-1^11^ secreted from the ectoderm have been implicated, and the LEM cells are thought to receive these chemokine signals via their respective receptors^8,12^. On the other hand, the LEM cells’ sensing of local mechanical interactions between the leader cells and follower cells has been reported to determine the asymmetric formation of cell protrusions and the oriented movement of LEM cells^13^. However, how such mechanical signals are interpreted by the cells remains unknown.

Intracellular Ca^2+^ signalling regulates a variety of physiological events, including cell proliferation, apoptosis, differentiation, and cell migration^14^. In cell migration, Ca^2+^ signalling regulates the effectors required for cytoskeletal remodelling and the establishment of focal adhesions^15–18^. Ca^2+^ signalling is essential both for the homeostasis of adult animals and for developmental processes such as organogenesis^19,20^. It is generally thought that intracellular Ca^2+^ signalling is regulated by fluctuations in the intracellular Ca^2+^ concentration, recognized as Ca^2+^ transients. There are two essential sources of intracellular Ca^2+^: influx from the extracellular space and release from the endoplasmic reticulum (ER). The influx of extracellular Ca^2+^ directly depends on cell membrane Ca^2+^ channels, while the release from the ER’s Ca^2+^ source depends on Ca^2+^ channels on the ER membrane^14^. The roles of these two modes of Ca^2+^ elevation in *Xenopus* development have been extensively studied. The Ca^2+^ transient and wave-like propagation of Ca^2+^ triggered by fertilization have been well characterized^21^, and this Ca^2+^ elevation is known to induce re-entry into the meiotic cell cycle^22^. In the gastrula stage, Ca^2+^ transients have been observed in the ectoderm and axial mesoderm^23,24^, suggesting that Ca^2+^ plays important roles in those tissues. Recent reports further indicate that Ca^2+^ signalling has critical roles in tissue morphogenesis^25–29^. Here we sought to clarify the intracellular Ca^2+^ dynamics and how they contribute to gastrulation cell movements. We first examined the Ca^2+^ dynamics of the migrating embryonic cells and the function of Ca^2+^ signals in the *Xenopus* LEM. We found that Ca^2+^ transients occur preferentially in the LEM cells during migration and are confined to the front rows of the LEM. Functional analyses in which the intracellular Ca^2+^ level was depleted by drug treatment and elevated with a Ca^2+^ ionophore demonstrated that the Ca^2+^ signal is necessary and sufficient for LEM migration. Finally, we found that Ca^2+^ transients are required for the polarized lamellipodia formation that accelerates LEM migration. Taken together, these results suggest that local Ca^2+^ signals in LEM cells contribute to the gastrulation cell movements of vertebrates.

## Results

### Intracellular Ca^2+^ transients in the leading edge mesoderm

**Figure 1:**
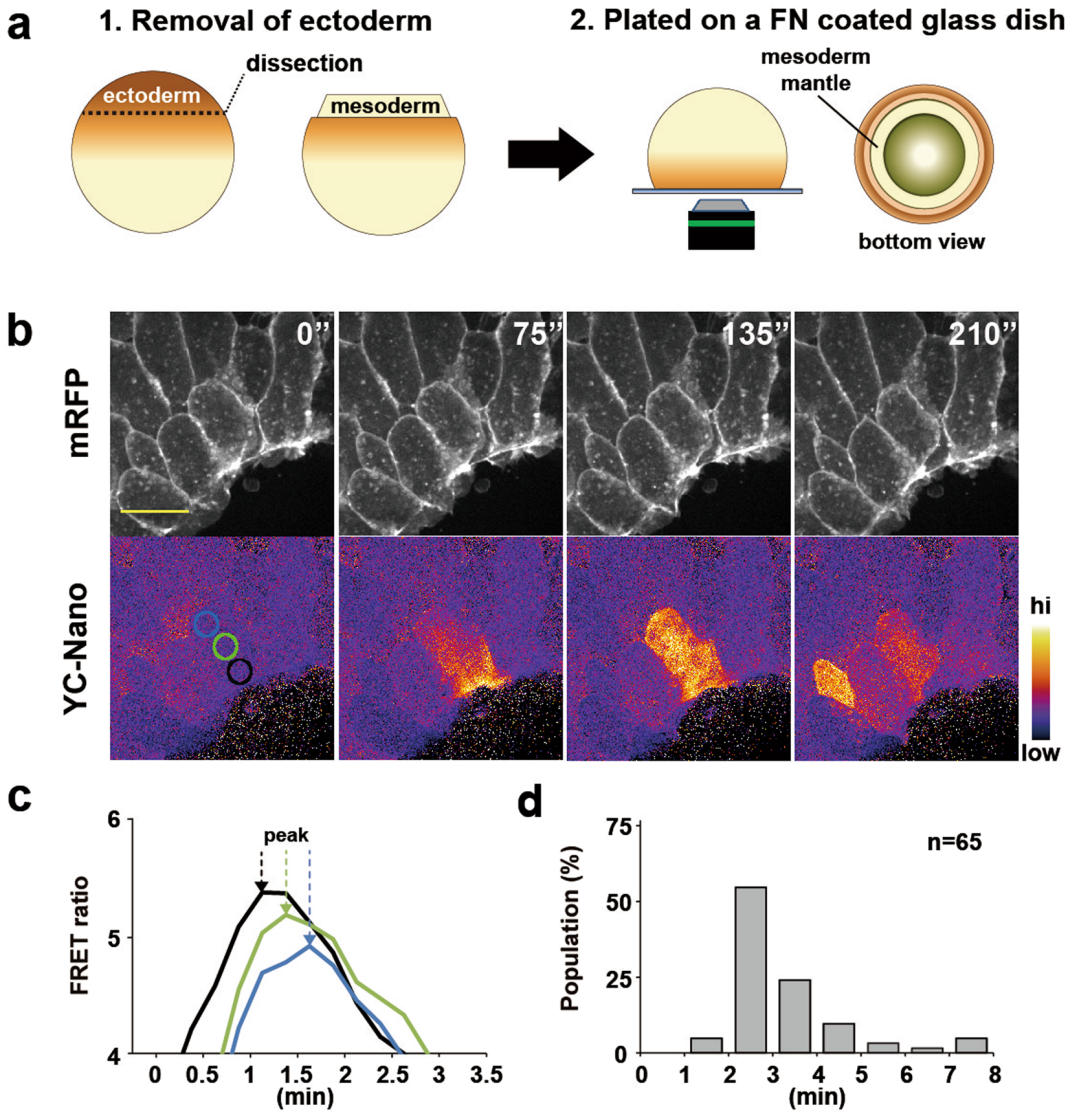
Ca^2+^ dynamics in a single cell. (**a**) Experimental design using cap-less explants. (1) The animal cap was removed at st12–12.5. (2) The cap-less explant was placed with the animal pole side down on a fibronectin-coated glass dish, and viewed from the bottom. (**b**) Snapshots from time-lapse calcium imaging of single cells. Upper panel: mRFP. Lower panel: FRET ratio of yellow cameleon-nano. The FRET ratio was converted to pseudocolours (bar at right). Scale bar: 50 μm. (**c**) Plot of the FRET ratio intensity over time for each of the areas shown in coloured circles in (**b**). Arrows indicate the points of maximum values. (**d**) Histogram of the calcium transient duration. n = 65 calcium transients.

First, to visualize the intracellular Ca^2+^ dynamics in LEM cells during gastrulation, we tested several variants of a FRET-based Ca^2+^ indicator yellow cameleon (YC)-Nano. We found that YC-Nano3GS^30^ had the most suitable dynamic range, enabling us to detect basal as well as transient increases in the intracellular Ca^2+^ of LEM tissue. To express the Ca^2+^ indicator and to label the cell membrane to visualize cell shape, we injected mRNAs for YC-Nano3GS and membrane-targeting RFP (mRFP), respectively, into the two dorsal blastomeres of 4-cell-stage embryos. However, there are well known technical limitations to observing cellular events in the gastrulating mesoderm, which is underneath the pigmented ectoderm. Therefore, to observe the cells undergoing gastrulation more clearly, we prepared “cap-less” embryos, as previously described (Fig. 1a)^3^. This preparation allowed us to observe the LEM cell movements occurring inside the embryo during gastrulation.

Time-lapse imaging of the cap-less embryo culture showed that the LEM underwent a directional migration toward the centre of the open field (the presumptive animal pole of a normal embryo) and finally ceased migrating soon after the open space was filled with cells, as previously reported. Using the Ca^2+^ probe YC-Nano3GS, we were able to observe the intracellular Ca^2+^ dynamics in LEM cells (Fig. 1b and Suppl. Movie 1). To characterize the Ca^2+^ dynamics at the single-cell level, we observed embryos at high magnification. At the single-cell level, the Ca^2+^ transients showed complex dynamics with varying durations and spatial patterns. The majority of the Ca^2+^ transients (76% of 59 transients from 2 embryos) in the LEM displayed wave-like diffusions at the subcellular level (Fig. 1b and c). These Ca^2+^ waves were initiated at the periphery of one side of the cell and travelled to the opposite side. Other Ca^2+^ transients diffused radially from the centre of cells to the periphery without waves (data not shown). Most transients lasted from 180–210 sec, although the duration ranged from 60 sec to several minutes (Fig. 1d). Thus, our live imaging of cap-less explants enabled us to detect the Ca^2+^ dynamics of migrating LEM cells at the subcellular level.

### Ca^2+^ transient dynamics in the leading-edge cells

**Figure 2:**
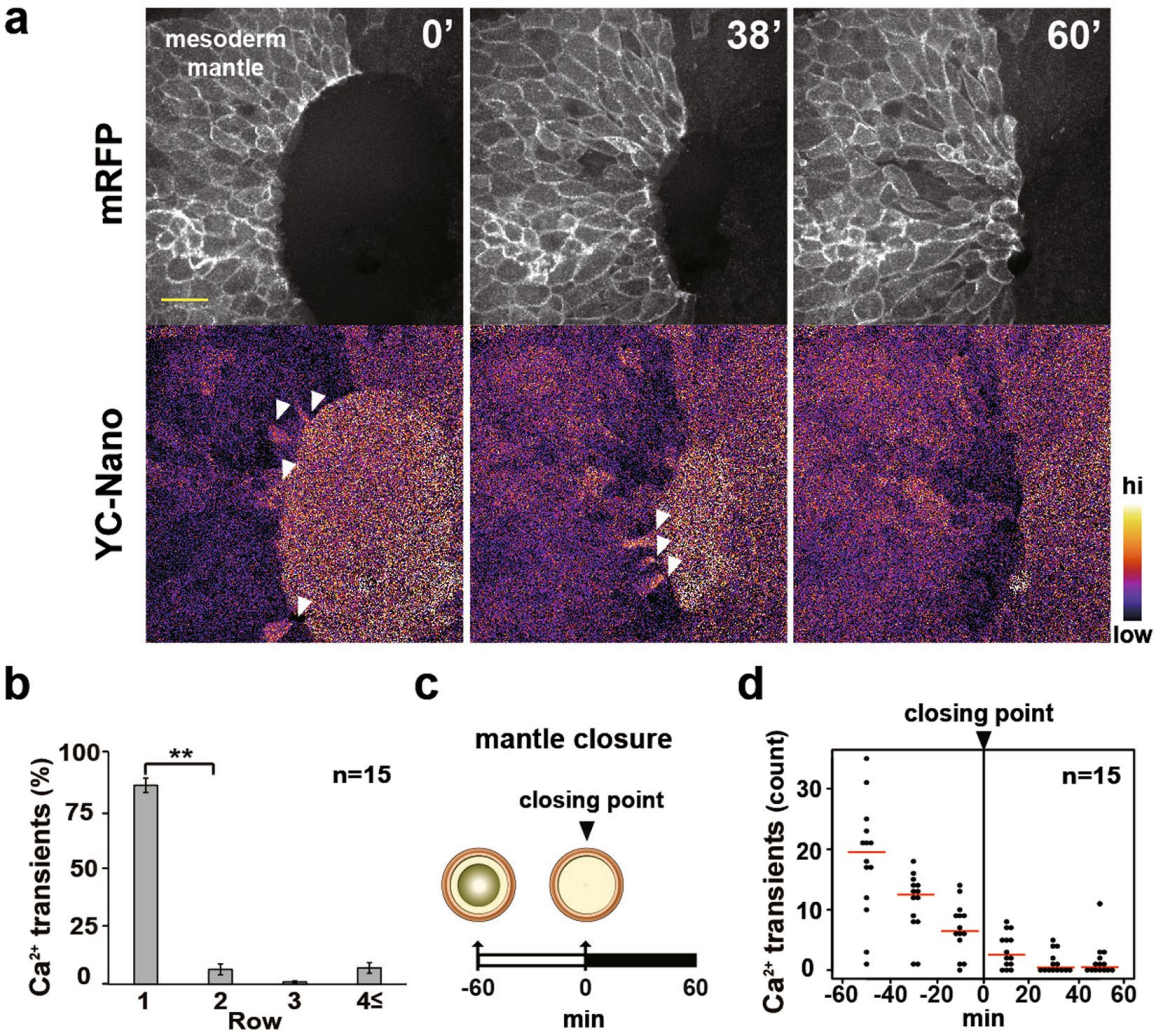
Ca^2+^ transients and their localization in LEM cells. (**a**) Snapshots of time-lapse imaging of the Ca^2+^ dynamics in the migrating LEM. FRET ratio images of yellow cameleon-nano were converted to pseudocolours (bar at right). White arrowheads indicate calcium transients. (**b**) Location of Ca^2+^ transients during mantle closing. n = 15 embryos. Error bars indicate s.e. ± Student’s t-test, **P < 0.005. (**c**) Illustration of the time course of mantle closure. (**d**) Frequency of Ca^2+^ transients during mantle closure. Red bars indicate average values. n = 15 embryos.

Next, we analysed the localization of calcium transients at the tissue level. Time-lapse imaging of the LEM showed that Ca^2+^ transients frequently occurred in the first row of LEM cells (Fig. 2a,b and Suppl. Movie 2), suggesting that they might have specific roles in cell migration. Next, we quantified the spatiotemporal dynamics of the Ca^2+^ transients during the gastrulation-like movement of the cap-less embryos by counting them automatically using Image J (Suppl. 1). Spatially, the leading cells in the first row showed a much higher frequency of Ca^2+^ transients than those in the second or third row (Fig. 2b). Interestingly, the incidence of Ca^2+^ transients in the LEM gradually decreased as the mantle closure proceeded (Fig. 2c and d). The frequency of Ca^2+^ transients was approximately one per minute at the beginning (about 20 transits from minutes 0–20) of cell migration, while Ca^2+^ transients were hardly detected after mantle closure (Fig. 2d). These observations suggested that the intracellular Ca^2+^ signal was functionally related to LEM migration.

### Ca^2+^ transient dynamics in DMZ explants

**Figure 3:**
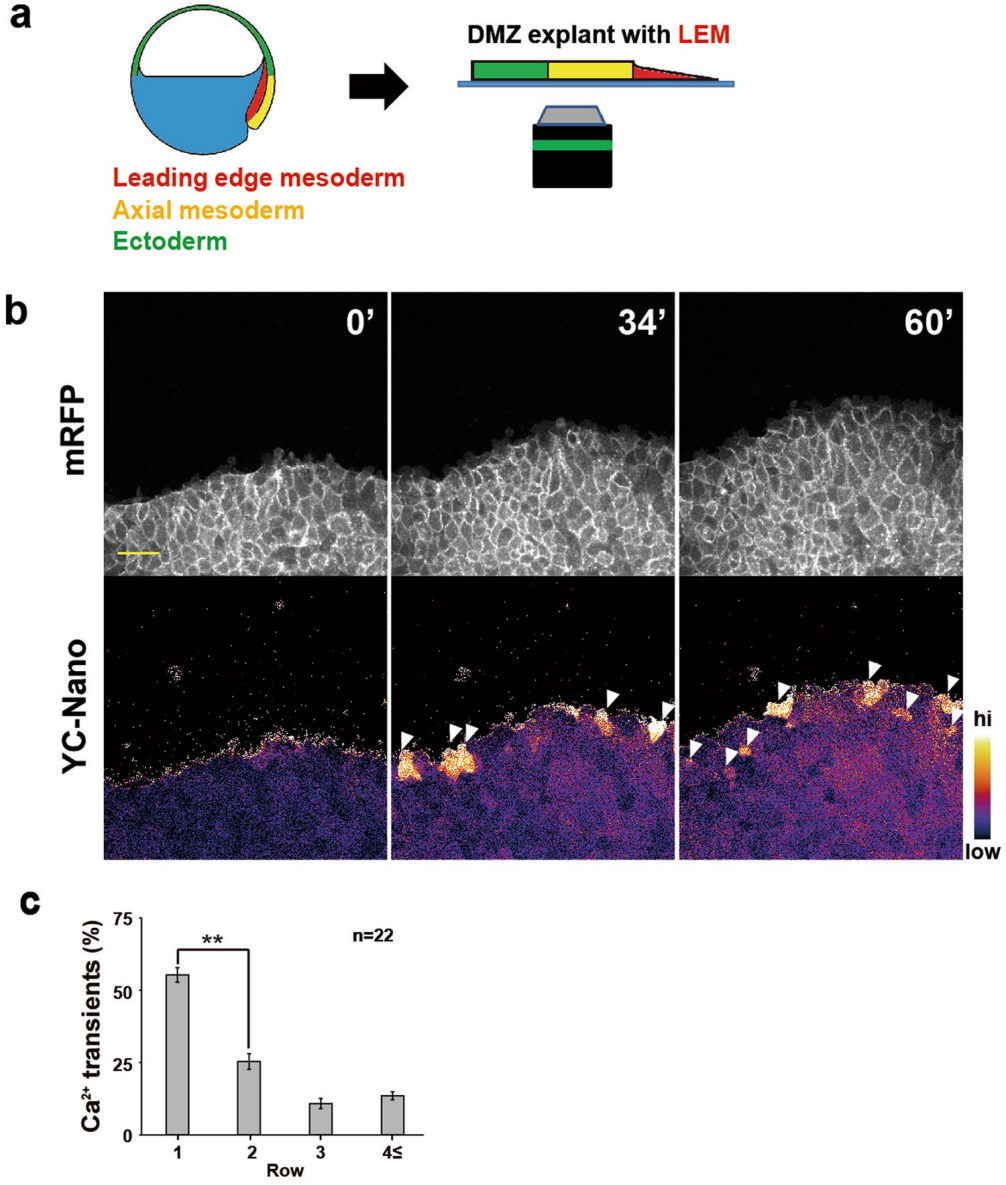
Ca^2+^ transients and their localization in DMZ explants. (**a**) Preparation of DMZ explants including the LEM. (**b**) Snapshots of time-lapse imaging of the Ca^2+^ dynamics in DMZ explants. The FRET ratio of yellow cameleon-nano was converted to pseudocolours (bar at right). White arrowheads indicate calcium transients. Scale bar: 100 μm. (**c**) Frequency of calcium transients during LEM migration in DMZ explants. n = 22 embryos Error bars indicate s.e. ± Student’s t-test, **P < 0.005.

The “cap-less” method is useful for observing the LEM in a quasi *in vivo* condition, but it can only reveal the LEM migration for a limited time, because it can only be applied to late-gastrula-stage embryos (around st12.5). Therefore, we next used dorsal marginal zone (DMZ) explants (Keller explants, Fig. 3a), which included the LEM and were excised from early-gastrula-stage embryos (around st11), and observed the leading edge migration for longer time periods (Fig. 3b and Suppl. movie 3). With the Keller explants, we observed similar Ca^2+^ transients that occurred preferentially in the first row of the LEM (Fig. 3c and Suppl. movie 3) although we also detected higher frequencies of transients in the following rows compared to the cap-less embryos. Similar to the cap-less embryos, most of the Ca^2+^ transients exhibited a wave-like pattern within the cell (Suppl. movie 4). These results suggested that Ca^2+^ transients occur throughout gastrulation, and that the high intracellular Ca^2+^ signalling activity in the leader cells plays an essential role in the regulation of cell behaviours during gastrulation.

### Intracellular Ca^2+^ transients are required for LEM migration

**Figure 4:**
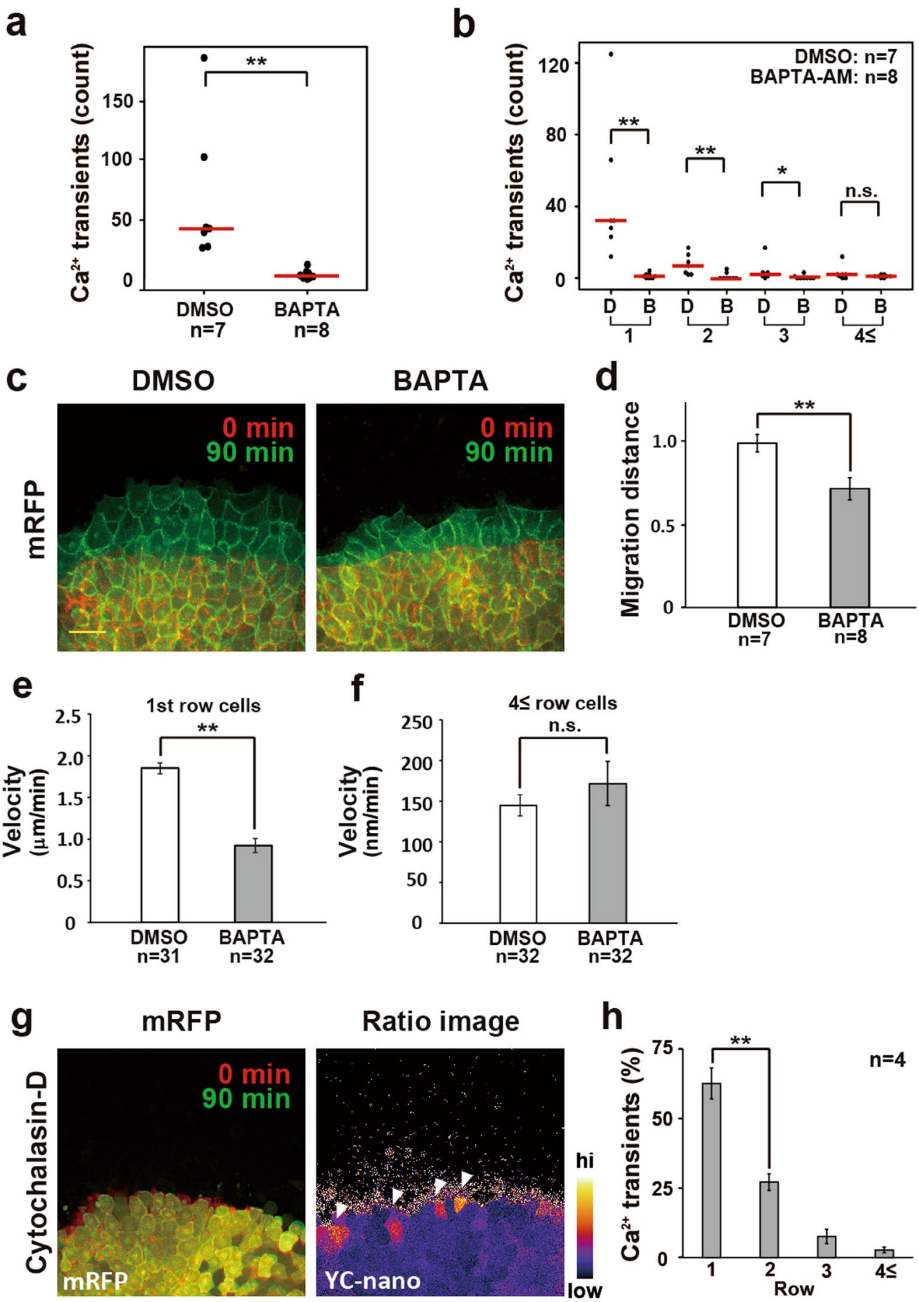
Ca^2+^ transients are required for LEM migration. (**a**) Ca^2+^ transients in DMZ explants treated with BAPTA-AM for 3 hours. Red bars indicate average values. DMSO: n = 7 embryos and BAPTA-AM (50 μM): n = 8 embryos. Mann–Whitney U-test, **P < 0.005. (**b**) Location of calcium transients during LEM migration in BAPTA-AM- or DMSO-treated DMZ explants. n = 8 embryos. Red bars indicate average values. Mann–Whitney U-test, **P < 0.005, *P < 0.05, n.s.: No significance. (**c**) Migration of DMZ explants treated with BAPTA-AM or DMSO. Migration was suppressed by BAPTA-AM treatment. Scale bar: 100 μm. (**d**) Relative migration distance in DMSO- or BAPTA-AM-treated DMZ explants. Values were normalized to the migration distance of DMSO-treated explants. Error bars indicate s.e. ± Student’s t-test, **P < 0.005. (**e**) Migration velocity of leader cells in DMSO- or BAPTA-AM-treated DMZ explants. Error bars indicate s.e. ± Student’s t-test, **P < 0.005. (**f**) Migration velocity of follower cells (≥4^th^) in DMSO- or BAPTA-AM-treated DMZ explants. Error bars indicate s.e. ± Student’s t-test, n.s.: No significance. (**g**) Left: Snapshot from time-lapse imaging. Cytochalasin-D (1 μM) treatment suppressed the migration activity in DMZ explants. Right: Ca^2+^ imaging of DMZ explants treated with Cytochalasin-D (1 μM). White arrowheads indicate Ca^2+^ transients. (**h**) Location of Ca^2+^ transients during 3 hours of Cytochalasin-D (1 μM) treatment. Error bars indicate s.e. ± Student’s t-test, **P < 0.005.

Next, we investigated the cellular function of the intracellular Ca^2+^ transients in the first few rows of the LEM of the DMZ during cell migration. To directly diminish the Ca^2+^ transients, we treated DMZ explants with a cell-permeable Ca^2+^ chelator, BAPTA-AM (50 μM), and observed the Ca^2+^ dynamics and the cell migration by time-lapse imaging of the leading region of the DMZ. BAPTA-AM-treated explants showed greatly reduced Ca^2+^ transients compared with DMSO-treated control explants (Fig. 4a and Suppl. movie 5). Quantitative analysis revealed that the BAPTA-AM treatment significantly reduced the Ca^2+^ transients particularly in the first row of the LEM and flattened the Ca^2+^ transient bias in the first few rows (Fig. 4b). These results indicated that the BAPTA-AM treatment efficiently reduced the intracellular Ca^2+^ transients in the LEM.

Next, we analysed the cell migration behaviour at the leading edge. Notably, the leading cells of the first row (Fig. 4c and d) in the BAPTA-AM-treated explants exhibited reduced migratory activity compared with those of DMSO-treated explants. We also measured cellular migration speed. BAPTA-AM-treatment reduced the leader cells’ migration speed, while the 4th or following cells were not significantly affected (Fig. 4e and f). We confirmed that neither cell-cell adhesion (Suppl. 2a) nor mesodermal differentiation was affected by the treatments (Suppl. 2b).

Next, we addressed the possibility that the Ca^2+^ transients were a consequence of cellular migration; for example, a result of cell stretching due to the movement of cohesive cells. To examine this possibility, we observed the Ca^2+^ dynamics in LEM immobilized by Cytochalasin-D treatment (Fig. 4f). Immobilizing the DMZ explants did not affect the Ca^2+^ transients at the leading edge (Fig. 4g and Suppl. movie 6), demonstrating that the Ca^2+^ transients were not the result of cellular migration. Taken together, these data indicated that Ca^2+^ transients at the leading edge are required for the normal cell migration of the DMZ.

### Forced increase in intracellular Ca^2+^ enhances the cell-migration capacity

**Figure 5:**
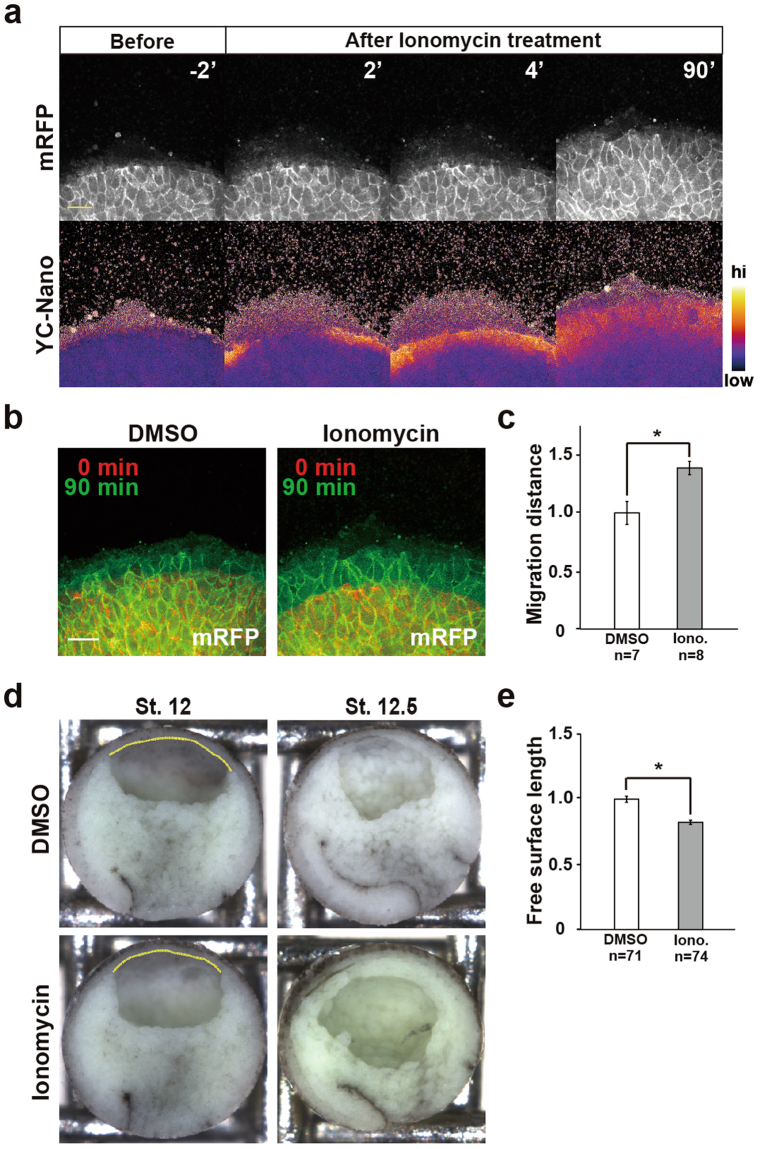
Intracellular Ca^2+^ signalling regulates migration activity in the LEM. (**a**) Snapshots from time-lapse imaging of the Ca^2+^ dynamics in DMZ explants treated with Ionomycin (2.5 μM). Upper panel: mRFP. Lower panel: FRET ratio of yellow cameleon-nano converted to pseudocolours (bar at right). (**b**) LEM migration into open space in Ionomycin- (2.5 μM) and DMSO-treated DMZ explants monitored by mRFP. (**c**) Relative migration distance in DMSO- and Ionomycin-treated DMZ explants. The migration distance was normalized to the migration distance of DMSO-treated DMZ. DMSO: n = 7 embryos. Ionomycin (Iono.): n = 8 embryos. Error bars indicate s.e. ± Student’s t-test, *P < 0.05. (**d**) Mid-sagittal section of whole embryos at st12 and 12.5. Left side: dorsal. Right side: ventral. Yellow line indicates blastocoel roof not touching mesoderm. (**e**) Graph of the measured length of the yellow line of Fig. 5d at st12.5. Values were normalized to the length in DMSO-treated Embryos. DMSO: n = 71 embryos. Ionomycin: n = 74 embryos. Error bars indicate s.e. ± Student’s t-test, *P < 0.05.

The above results suggested that the Ca^2+^ signal at the leading edge promotes cell migratory activity. To test this hypothesis, we stimulated intracellular Ca^2+^ signalling by applying the Ca^2+^ ionophore Ionomycin to the DMZ explants (Fig. 5a). The Ionomycin treatment induced higher migratory activity compared with DMSO-treated control explants (Fig. 5b and c). Next, we examined whether Ionomycin treatment could facilitate gastrulation cell movements, particularly the anterior migration, in embryos. Notably, the Ionomycin-treated embryos showed accelerated LEM migration when examined by bisectioning at st.12 (Fig. 5d and e), compared with control embryos. At st.13, when mesodermal mantle from the ventral and dorsal side simply met at the animal pole in the control embryo, in the Ionomycin-treated embryos the migrating mesodermal sheets from the ventral and dorsal sides not only met but also overrode each other at the animal pole (Fig. 5d). Like BAPTA-AM, Ionomycin-treatment affected neither cell-to-cell adhesion (Suppl. 2a) nor mesodermal differentiations (Suppl. 2). These results suggested that Ca^2+^ signalling positively regulates the LEM migration during gastrulation.

### Disruption of Ca^2+^ transients results in abnormal cell protrusive activity

**Figure 6:**
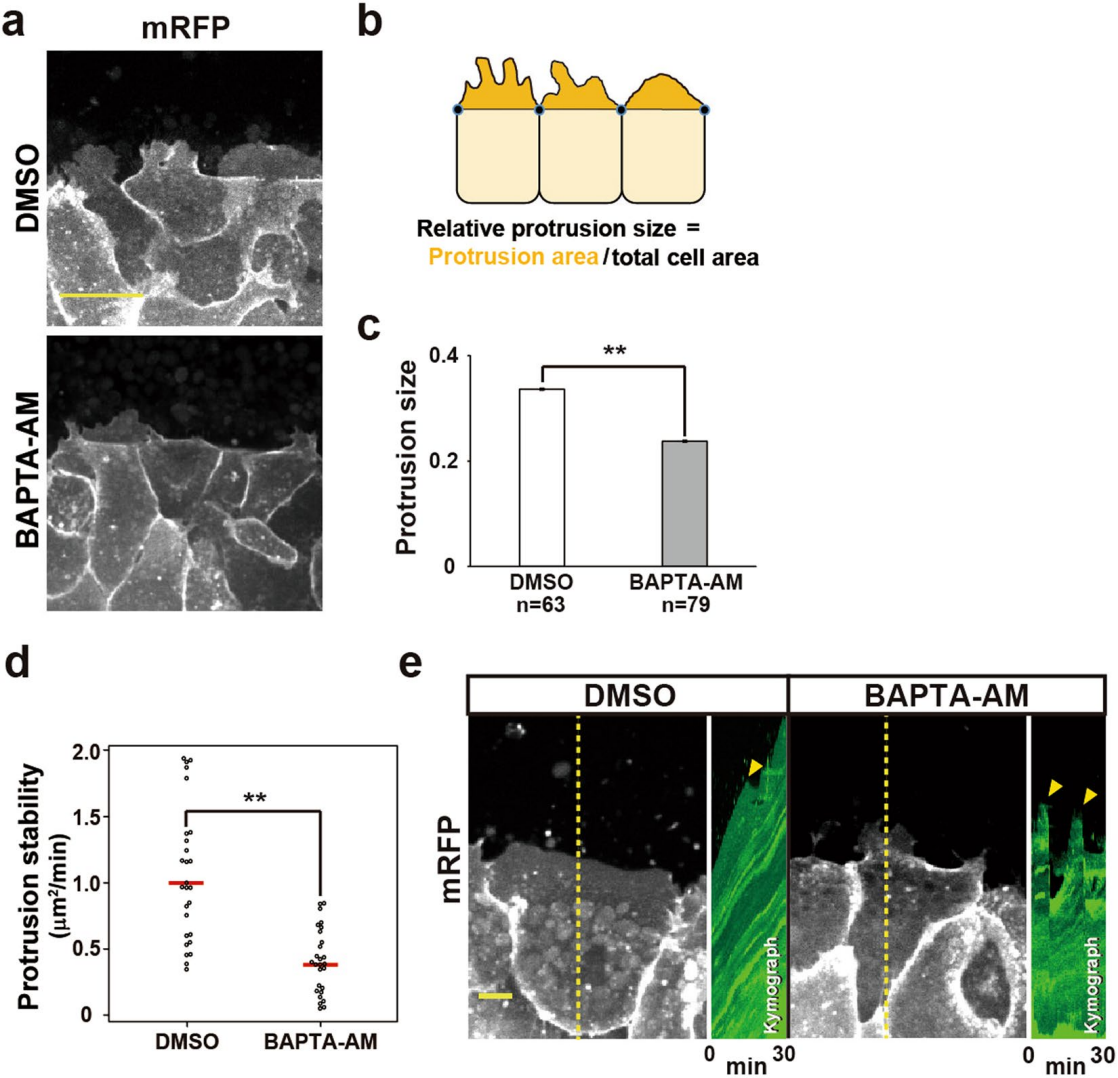
Suppression of Ca^2+^ transients reduce the protrusive activity in LEM cells. (**a**) Snapshots of BAPTA-AM- and DMSO-treated DMZ explants. Scale bar: 50 μm. (**b**) Procedure for measuring the protrusion activity in LEM cells. (**c**) Protrusion size in DMSO- and BAPTA-AM-treated LEM cells. DMSO: n = 63 cells from 14 embryos, BAPTA-AM: n = 79 cells from 13 embryos. Error bars indicate s.e. ± Mann–Whitney U-test, **P < 0.005. (**d**) Protrusion stability in DMSO- and BAPTA-AM-treated LEM cells. DMSO: n = 27 cells from 9 embryos, BAPTA-AM: n = 27 cells from 9 embryos. Red bars indicate average value. Student’s t-test, **P < 0.005. (**e**) Protrusion dynamics in DMSO and BAPTA-AM-treated LEM cells. Left figure with yellow dotted line is entire view of leader cell. Right figure is kymograph along to dotted yellow line. Yellow arrowheads indicate retraction of cellular protrusion. Scale bar: 25 μm.

Together, these results demonstrated that intracellular Ca^2+^ signalling regulates cell migration processes. We next investigated how the Ca^2+^ signalling affected the leading edge mesodermal cells, by carefully analysing the cellular morphology of control and BAPTA-AM-treated explants. During the gastrulation of normal explants, most leading edge cells display active protrusions into the free surface area^3^. We digitally segmented the areas of the cell protrusions of DMSO- and BAPTA-AM-treated explants in microscopy images and compared them quantitatively. In DMSO-treated explants, the protrusions extended far into the free surface area, similar to those seen in intact embryos (Fig. 6a, upper panel). In BAPTA-AM-treated explants, cellular protrusions extending into the free surface were also observed (Fig. 6a, bottom panel). However, quantitative analysis (Fig. 6b) showed that the cellular protrusion areas were significantly smaller in the BAPTA-AM-treated cells than in the DMSO-treated ones (Fig. 6c). We further quantitated cellular protrusion dynamics by measuring the temporal change of protrusion size. The average protrusion size per minute over 30 min is significantly reduced (Fig. 6d). The kymograph showed that BAPTA-AM-treated explants occasionally exhibited rapid retraction of its protrusion (Fig. 6e). These observations led us to speculate that the Ca^2+^ transients regulate the cell migratory activity by controlling the cellular protrusion activity.

### Ca^2+^ signalling regulates Rac1 activity

**Figure 7:**
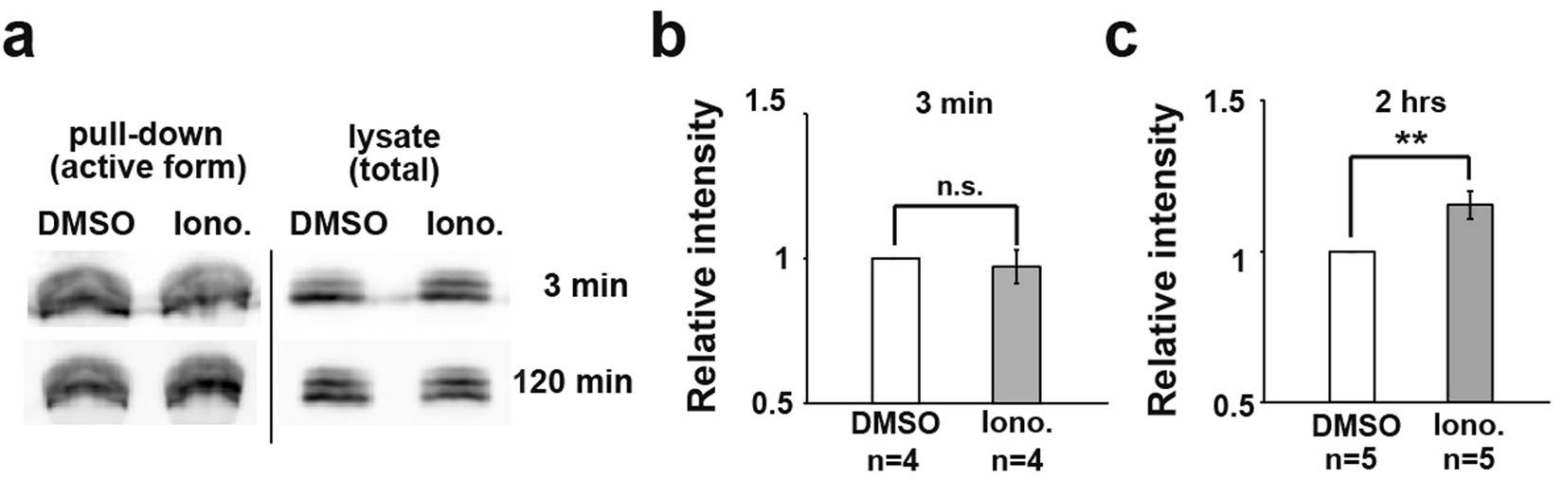
Ca^2+^ signalling regulates Rac1 activity. (**a**) Rac1 activity after Ionomycin (Iono.) treatment for 3 minutes and 2 hours. DMZ explants were dissected and treated with Ionomycin. DMSO was used as a control. (**b**) Rac1 activity after Ionomycin treatment for 3 minutes. The active Rac1 intensity with Ionomycin was normalized to the intensity in DMSO-treated samples. Each western sample was prepared from 35–40 DMZ explants. Error bars indicate s.e. ± Student’s t-test, ns: No significance. (**c**) Rac1 activity after Ionomycin treatment for 2 hours. Student’s t-test, **P < 0.005.

We next examined how the Ca^2+^ dynamics regulate cell migration activity in the LEM with respect to the downstream components of the Ca^2+^ signalling. Among the pathways activated by Ca^2+^ signaling^18,31,32^, Rac is a small GTPase that is known to be essential for forming the lamellipodia that are observed on migrating LEM cells during *Xenopus* gastrulation^3^. Rac signalling is also reported to be involved in LEM cell migration^10^. Therefore, we next investigated whether a single Ca^2+^ transient or continuous and repeated transients could activate Rac signalling. To quantify Rac1’s GTPase activity, we expressed myc-tagged Rac1 in the dorsal side of the embryo by micoinjecting its mRNA into the two dorsal blastomeres at the 4-cell stage. When the embryo reached st.12, the active form of Rac1 was quantified by a pull-down assay with PAK-PBD, which binds the active form of Rac/Cdc42. DMZ explants treated with Ionomycin for a 3-minute period (the average duration of a Ca^2+^ transient), representing a single transient, or for 2 hours, representing continuous and repeated Ca^2+^ transients, were subjected to the pull-down assay (Fig. 7a), and the band intensities were quantified and compared. While the 3-minute treatment did not increase the amount of active Rac1 (Fig. 7b), the 2-hour treatment significantly increased it (Fig. 7c). These results indicated that although a single Ca^2+^ transient is not sufficient to induce Rac1 activation, a prolonged Ca^2+^ signal for 2 hours can enhance the Rac1 activity.

## Discussion

In this study, we used *Xenopus* gastrulation as a model of massive tissue movements, and analysed its Ca^2+^ dynamics by live imaging. We observed constant Ca^2+^ transients preferentially in the leading edge, especially in the first few rows. We also found that these Ca^2+^ transients were strongly correlated with LEM migration. An intracellular Ca^2+^-specific chelator (BAPTA-AM) reduced the migratory activity of the LEM. Conversely, Ca^2+^ induction by a Ca^2+^ ionophore (Ionomycin) accelerated the migratory activity of the LEM. Furthermore, we found that Ca^2+^ elevation enhanced the Rac1 activity. Taken together, these results suggested that Ca^2+^ signalling at the edge of the LEM regulates the migration activity of the tissue and therefore controls gastrulation cell movements.

To visualize Ca^2+^, we used a FRET-based Ca^2+^ indicator YC-nano 3GS, and observed the LEM by spinning disc confocal microscopy. This indicator is suitable for measuring the steady-state concentrations^30^ of intracellular Ca^2+^and enabled us to observe and characterize the Ca^2+^ transients in detail under various experimental conditions. We found that almost all of the Ca^2+^ transients occurred in single cells, and only occasionally did we observe wave-like propagations over multiple cells in the leading mesodermal cells (data not shown) as previously described^28^. In the leading edge, most of the Ca^2+^ transients had a duration of 180 to 210 sec. These rather long durations differ from those reported for *Xenopus* mesodermal cells (90 sec)^19,28^ and neural plate (less than 40 sec)^26,27^. At the subcellular level, temporal analysis of the Ca^2+^ dynamics revealed that almost all of the Ca^2+^ transients showed a wave-like pattern (Fig. 1b and c). Ca^2+^ transients are known to occur responding to wounds^33,34^. Therefore, we compared the dynamics of Ca^2+^ transients we observed is similar to those in wound responses. Immediately after the tissue injury by a fine needle, massive calcium transients over the cells occurred as previous described^33,34^ (data not shown). However, this profile is distinct from calcium transients we observed which are restricted to few cells. Because our explants and cap-less embryo were observed 60 min after manipulation, it is unlikely that the Ca^2+^ transient is a direct wound response.

However, we were not able to address what actually triggers the calcium transients in the LEM. Notably, these wave-like patterns are reported in various cells^35,36^, and are commonly stimulated by the calcium influx from efflux from ER store; therefore, one of our speculations is that RyR receptor or IP3 receptor are involved in triggering the Ca^2+^ transients in the LEM. Other sources include extracellular space^37^, mitochondoria^38^ and lysosomal calcium efflux in which acidic calcium store of lysosome regulates calcium transient like ER store^39^.

In our experiments, suppressing the Ca^2+^ transients reduced the cellular protrusions of the leader cells (Fig. 6c) and accordingly, compromised the cell migration of mesodermal sheets (Fig. 4d). Conversely, the forced Ca^2+^ elevation by Ionomycin treatment increased the cell-migrated area (Fig. 5c). These data demonstrated that Ca^2+^ transients are necessary and sufficient for LEM migration, and that Ca^2+^ signalling positively regulates the migratory activity.

Regarding how Ca^2+^ dynamics regulate the migration activity, we demonstrated that forced Ca^2+^ signalling increased the Rac1 activity, a known regulator of cell protrusion formation. Notably, a single transient Ca^2+^ stimulation for a short period (3 minutes) was not sufficient to activate Rac1 activity, but continuous Ca^2+^ stimulation did activate it (Fig. 7b and c). These results demonstrated that an intracellular Ca^2+^ elevation could activate Rac1 activity, although a missing link remains between the Ca^2+^ dynamics and Rac1 activation. In this study, we also attempted to test the effect of brief calcium transients with caged-IP3 (Suppl. 3) on cellular protrusion by reproducing a single transient. Although we were able to synthesize a calcium transient for 3 min in a group of cells, we failed to confirm that the protrusive activity was enhanced after the 3-minute synthesized Ca^2+^ transient, suggesting that a single Ca^2+^ transient may not be sufficient to activate the cellular protrusion activity. Taking these results together, we propose two possible models for the duration of Ca^2+^ transients and migratory activity. One is a “delayed response model,” which suggests that the cellular response to a Ca^2+^ transient takes longer than the transient (at least more than 20 min). The other is an “accumulation model,” which suggests that repeated Ca^2+^ transients induce cellular protrusion activity when the accumulated signal reaches a certain threshold.

We found that leader cells showed the highest frequency of Ca^2+^ transients during gastrulation (Fig. 2a and d), which in turn suggested that even in the collectively migrating LEM cells, cells at different positions have different properties. It was previously reported that the edge cells of *Xenopus* LEM show condensed actin polymerization in their cell protrusions^3^. It was also recently shown that the leader cells have unique roles in collective migration^40–42^. Our present study suggests that Ca^2+^ transients might confer the cells a leader identity through the activation of Rac1. This is true for the Ionomycin-treated whole embryos (Fig. 5) and the increased Ca^2+^ and Rac1 activity in the explant promoted the cell migration efficiently.

We also found that the calcium transients ceased after mantle closure in cap-less explants, while DMZ explants continuously showed calcium transients. This apparent difference may indicate that the calcium transients are ceased by the cell’s touching of another cell mass. Although immobilizing cells with Cytochalasin-D did not affect the Ca^2+^ transients in the leader cells (Fig. 4e and f), physical cell-cell interaction might provide the signal to stop Ca^2+^ transients in these cells.

In this study, we focused on cellular migration activity, and our findings suggested that calcium signalling regulates Rac1 activity. However, we explored only one Ca^2+^ function in LEM migration, among various Ca^2+^-activated pathways. To better understand the role of calcium signalling in cell migration in general, we need to establish an overall view of the signalling pathways triggered by a large repertoire of Ca^2+^ dynamics.

## Methods

### Ethics statement

All of the protocols for animal experiments and animal care were approved by the Institutional Animal Care and Use Committee of the National Institutes of Natural Sciences, Japan. All of the experimental animal procedures were performed according to the guidelines of the institutions.

### mRNA synthesis

For *in vitro* transcription, plasmids were linearized with NotI. Capped mRNAs were synthesized using the mMESSAGE mMACHINE SP6 kit (Ambion), and purified with a NICK column (Pharmacia). The YC-nano^30^ and membrane-tagged RFP (mRFP) constructs were previously reported^43^.

### RT-PCR

For RT-PCR with drug-treated DMZ explant, 5 explants were dissociated at St. 10.5 and incubated with various drug for 3 hours. The following primers were used: ODC, Cerberus, Xbra and Xnot^44,45^.

### Microinjection

Female adult *Xenopus laevis* were induced to ovulate by injecting human gonadotropin. The collected eggs were fertilized *in vitro*, treated with 3% cysteine for dejellying, and reared in water. For microinjection, embryos were placed in a solution of 0.3% Ficoll in 1x MMR. mRNAs and NPE-caged IP3 were injected into the dorsal region at the four-cell stage. Injected embryos were reared in the same buffer until the appropriate stages.

### Chemical treatment

To treat whole embryos, Ionomycin (095–05831, Wako) in DMSO was added to the medium at st. 11. To treat explants, Ionomycin in DMSO was added to the medium by pipet during live imaging. BAPTA-AM (B035, Dojindo) in DMSO was added to the medium 30 minutes before starting the live imaging.

### Explant preparation

To prepare cap-less explants, ectoderm was removed from the embryo at the late-gastrula stage (st. 12–12.5), as previously described^3^. The cap-less embryo was plated on a fibronectin (FN)-coated glass-bottom dish. For DMZ explants with LEM, at the early gastrula stage (st. 11–11.5), a 60–80° dorsal marginal zone (DMZ) explant including the LEM was dissected by tungsten needle and immediately mounted onto a FN-coated glass-bottom dish. These mounted explants were incubated for at least 1 hour before microscopic observation. All explants were cut and incubated in Danilchik’s for Amy (DFA) plus bovine serum albumin (BSA) medium^46^.

### Western blotting for active Rac1

To measure Rac1 activity, we used the Rac1 Pulldown Activation Assay Biochem Kit (BK035, Cytoskeleton, Inc.). DMZ explants expressing myc-tagged xlRac1 were treated with Ionomycin for 3 minutes or 2 hours. To detect Rac1 activity, after Ionomycin or DMSO treatment in DFA medium, the DMZ explants were washed with 1 ml of Steinberg’s solution. The collected explants were lysed in lysis buffer (50 mM Tris pH 7.5, 10 mM MgCl2, 0.5 M NaCl, and 2% Igepal) with a cocktail of protease inhibitors. The supernatant of the lysed sample was collected and used for a pull-down assay with GST-tagged PAK-PBD beads, which bind to active Rac1. The samples were denatured by adding an equivalent volume of 2x SDS sample buffer (0.5 M Tris–HCl pH 6.8, 10% SDS, 50% glycerine, 5% 2-mercaptoethanol). After boiling for 5 min, the samples were subjected to SDS-PAGE and blotted onto PVDF membranes (Bio- Rad). For detection, an anti-myc antibody (#2272 Cell Signaling), HRP-conjugated secondly antibody, and ECL kit (GE Healthcare) were used.

### Calcium imaging

To analyse the calcium dynamics, 500 μg of YC-nano 3GS mRNA was dorsally injected into 4-cell-stage embryos. All calcium imaging was done using an inverted microscope (IX81, Olympus) equipped with a spinning disk confocal unit (CSU-X1, Yokogawa), EMCCD camera (iXon3, Andor), and 445-, 488-, and 561-nm lasers (Andor). All of the images were obtained with 100–500-ms scanning every 15–120 seconds for 0.5–3 hours at 20 degrees Celsius with a 10x (UPlanSAPO 10x/0.4, Olympus) or 20x (UPlanAPO 20x/0.7, Olympus) objective lens.

### Un-caging experiment

To provide the UV illumination for uncaging experiments, a mercury lamp-based light source with a band-pass filter (AT350/50x; Chroma) was installed on the spinning disk confocal system, with a shutter controlled by iQ2 software (Andor). To uncage the NPE-Caged IP3, the explants were illuminated for 3 seconds with UV light.

### Image analysis of calcium dynamics

Images were analysed using Image J software. The ratio between the YFP and CFP of YC-nano was calculated after background subtraction. We used the average intensity of an empty sample region as the background. A calcium transient was defined as an increase in the ratiometric value greater than the moving median image of the ratiometric value (Suppl. 1).

### Cell migration analysis

For cell migration analysis, we measured three different ordinates (rightmost, middle, and leftmost) in the leader cells at three time points (0 min, 90 min, and 180 min). We measured the migration distance for 180 min at the three different ordinates and calculated the average of the three distances as the migration activity.

For the measurement migration velocity, we monitored the displacement of cell position from the start (0 min) to the end points (90 min) and velocity was calculated by dividing the distance by 90 min.

### Analyses of cellular protrusion stablity

To quantitate celllular protrusion stability, we measured membranous protrusion areas over 30 min and calculate the average protrusion area per minute as the protrusion stability.

### Data Availability

All our data in this article will be provided upon request.

## Electronic supplementary material

Supplementary figures and figure legends

Supplementary movie 1

Supplementary movie 2

Supplementary movie 3

Supplementary movie 4

Supplementary movie 5

Supplementary movie 6

Supplementary movie 7
