# Supplementary material for: Intracellular calcium signal at the leading edge regulates mesodermal sheet migration during Xenopus gastrulation
Source: Sci Rep. 2018 Feb 5;8:2433. doi: 10.1038/s41598-018-20747-w (PMC5799360; doi:10.1038/s41598-018-20747-w)
Supplement: Supplementary file 1 — Supplementary figures and figure legends [file 41598_2018_20747_MOESM1_ESM.pdf]

Supplementary Information

Intracellular calcium signal at the leading edge regulates mesodermal sheet migration during *Xenopus* gastrulation

Kentaro Hayashi[1,2], Takamasa S. Yamamoto[1] and Naoto Ueno[1,2,*]

1. Department of Developmental Biology, National Institute for Basic Biology, 38 Nishigonaka, Myodaiji, Okazaki, Aichi 444-8585, Japan
2. Department of Basic Biology, School of Life Science, The Graduate University of Advanced Studies (SOKENDAI), 38 Nishigonaka, Myodaiji, Okazaki, Aichi 444-8585, Japan

   * Email: nueno@nibb.ac.jp

Supplementary figure 1

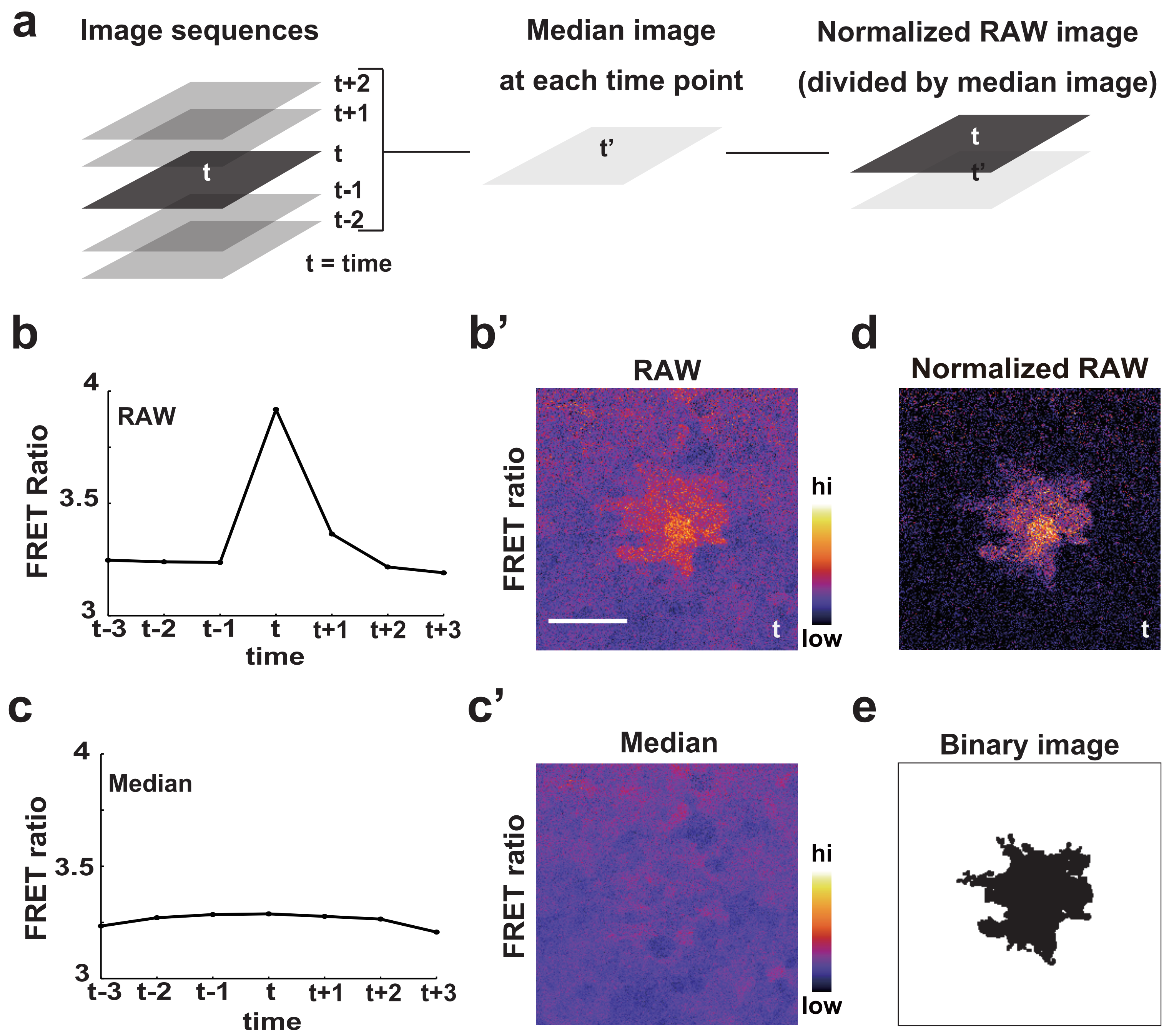

Supplementary figure 2

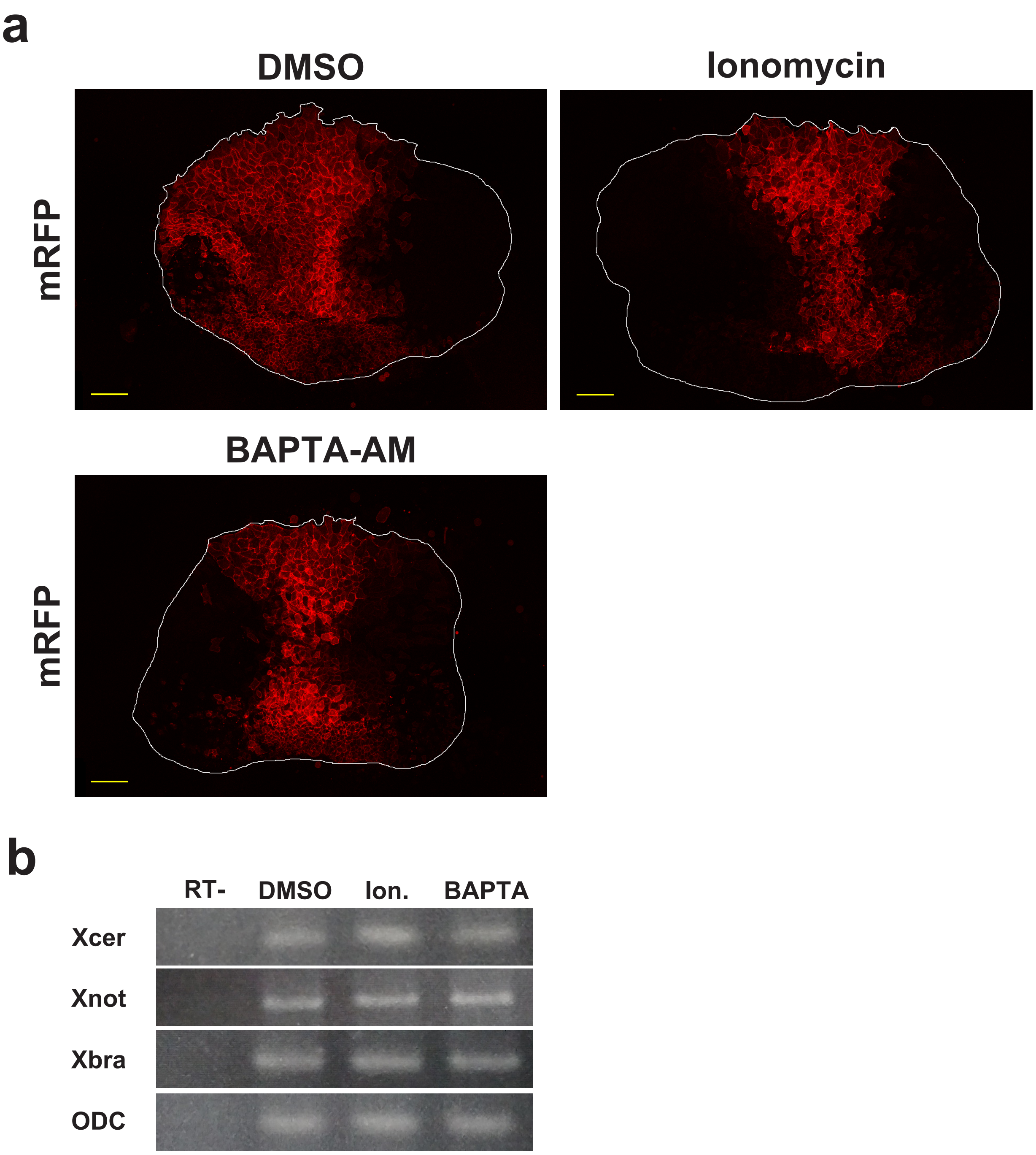

Supplementary figure 3

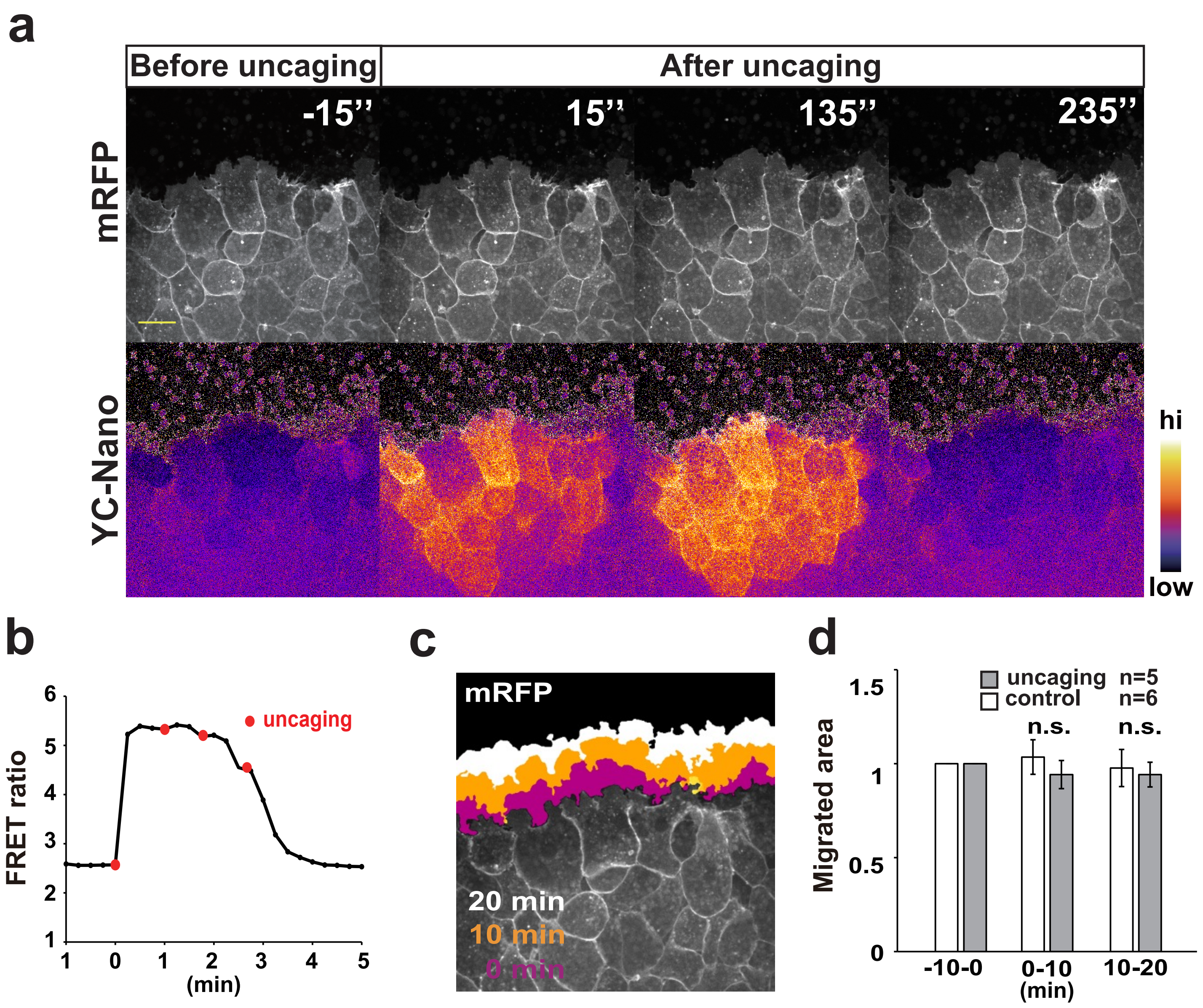

Supplementary figure and movie legends

Suppl. 1 Image analysis of Ca^{2+} transients

a. Procedure for the image analysis of Ca^{2+} transients. Left and Middle: A "moving median image" was obtained from the RAW image sequences over time. Right: To detect calcium transients, the RAW image was divided by the moving median image.

b. Plot of the FRET ratio of Ca^{2+} transients from the RAW image sequences over time.

b'. FRET image of a Ca^{2+} transient. Scale bar: 100 μm.

c. Photo intensity from the moving median image sequences in B.

c'. Moving median image of B'.

d. Normalized RAW image (B' image divided by C' image).

e. Binary image divided by the C' image.

Suppl. 2 Effects for cell-cell adhesion and mesodermal differentiation by drug treatments

a. Snapshots of drug-treated DMZ explants. White line indicates outline of explants. Scale bar: 250 μm

b. RT-PCR of mesodermal markers.

Suppl. 3 LEM migration activity after uncaging caged-IP3

a. Snapshots of photo-inductive Ca^{2+} transients. Upper panels: mRFP. Lower panels: FRET ratio image of yellow cameleon-nano. Scale bar: 50 μm

b. FRET ratio change by a photo-induced calcium transient.

c. Migration activity of the LEM. Time-lapse images at 0 min (magenta), 10 min (orange), and 20 min (white).

a. Graph of the migrated area at each period. Values were normalized to the migrated distance during the -10-0 min period. Error bars indicate s.e. ±. Student's t-test, ns: No significance.

Suppl. movie 1: Wave-like Ca^{2+} transients in the leader cells in a cap-less embryo.
Ca^{2+} dynamics of LEM cells in a cap-less embryo. Left:mRFP. Right:Ratio image of YC-nano 3GS. 20x objective lens.

Suppl. movie 2: Ca^{2+} transients in the migrating mesoderm in a cap-less embryo.
Ca^{2+} transients of LEM in a cap-less embryo. Left:mRFP. Right:Ratio image of YC-nano 3GS. 10x objective lens.

Suppl. movie 3: Ca^{2+} transients in a DMZ explant.
Ca^{2+} transients of LEM in a DMZ explant. Left:mRFP. Right:Ratio image of YC-nano 3GS. 10x objective lens.

Suppl. movie 4: Wave-like Ca^{2+} transients of leader cells in a DMZ explant.
Wave-like Ca^{2+} transients of leader cell in DMZ. Left:mRFP. Right:Ratio image of YC-nano 3GS. 20x objective lens.

Suppl. movie 5: Ca^{2+} transients after BAPTA-AM treatment in a DMZ explant.
Ca^{2+} dynamics of LEM in a DMZ explant after BAPTA-AM treatment. Left:mRFP. Right:Ratio image of YC-nano 3GS. 10x objective lens.

Suppl. movie 6: Ca^{2+} transients after Cytochalasin-D treatment in a DMZ explant.
Ca^{2+} dynamics of LEM in a DMZ explant after Cytochalasin-D treatment. Left:mRFP. Right:Ratio image of YC-nano 3GS. 10x objective lens.

Suppl. movie 7: Ca^{2+} transients after uncaging caged-IP3
Ca^{2+} transient of LEM in restricted cells before/after uncaging caged IP3. Left:mRFP. Right:Ratio image of YC-nano 3GS. 20x objective lens.
